# Identification of CD4^−^CD8^−^ Double-Negative Natural Killer T Cell Precursors in the Thymus

**DOI:** 10.1371/journal.pone.0003688

**Published:** 2008-11-10

**Authors:** Nyambayar Dashtsoodol, Hiroshi Watarai, Sakura Sakata, Masaru Taniguchi

**Affiliations:** Laboratory for Immune Regulation, RIKEN Research Center for Allergy and Immunology, Tsurumi-ku, Yokohama, Kanagawa, Japan; Karolinska Institutet, Sweden

## Abstract

**Background:**

It is well known that CD1d-restricted Vα14 invariant natural killer T (NKT) cells are derived from cells in the CD4^+^CD8^+^ double-positive (DP) population in the thymus. However, the developmental progression of NKT cells in the earlier stages remains unclear, and the possible existence of NKT cell presursors in the earlier stages than DP stage remains to be tested.

**Principal Findings:**

Here, we demonstrate that NKT cell precursors that express invariant *Vα14-Jα18* transcripts but devoid of surface expression of the invariant Vα14 receptor are present in the late CD4^−^CD8^−^ double-negative (DN)4 stage and have the potential to generate mature NKT cells in both *in vivo* and *in vitro* experimental conditions. Moreover, the DN4 population in CD1d knock-out (CD1dKO) mice was similar to those with an NKT cell potential in wild-type (WT) C57BL/6 (B6) mice, but failed to develop into NKT cells *in vitro*. However, these precursors could develop into NKT cells when co-cultured with normal thymocytes or in an *in vivo* experimental setting, indicating that functional NKT cell precursors are present in CD1dKO mice.

**Conclusions:**

Together, these results demonstrate that thymic DN4 fraction contains NKT cell precursors. Our findings provide new insights into the early development of NKT cells prior to surface expression of the invariant Vα14 antigen receptor and suggest the possible alternative developmental pathway of NKT cells.

## Introduction

The developmental progression of the T cell lineage in the thymus is precisely controlled, beginning with the early T cell precursors, which present in the population of lineage (Lin)^−^, CD4^−^CD8^−^ double-negative (DN) thymocytes. The DN population in the thymus can be further divided based on their expression of CD25 and CD44 into DN1 (CD25^−^ CD44^+^), DN2 (CD25^+^ CD44^+^), DN3 (CD25^+^ CD44^−^) and DN4 (CD25^−^ CD44^−^), which are sequential developmental stages [1]. Major commitment events to the αβ T cell lineage occur at the DN3 stage, where rearrangement of the T cell receptor (TCR) β chain gene (*Tcrb*) and subsequent beta-selection for a functional TCRβ chain take place. The cells that survive beta-selection develop into the DN4 stage to become CD4^+^CD8^+^ double-positive (DP) thymocytes. In the case of conventional αβ T cells, rearrangement of the TCRα chain gene locus occurs at the DP stage, and those cells that make productive rearrangements undergo MHC-mediated positive and negative selection to generate single-positive (SP) CD4 or CD8 T cells [2].

Natural killer T (NKT) cells are characterized by the expression of an invariant antigen receptor encoded by *Vα14-Jα18* in mice and *Vα24-Jα18* in humans. This receptor is used preferentially by NKT cells but not by conventional T cells, defining NKT cells as a distinct lineage from conventional T cells [3]. NKT cells recognize self- or non-self glycolipid ligands in conjunction with the monomorphic MHC-like molecule CD1d [4], and mediate intermediary functions that link the innate and acquired immune systems, regulating protective and regulatory responses by their rapid secretion of large amounts of cytokines such as IL-4, and IFN-γ after activation [5], [6].

Since the discovery of this unique cell lineage, the developmental pathway of NKT cells has been one of the most intriguing topics. Based on previous findings that cells with NKT cell potential can be detected in the DP thymocyte population [7], [8], NKT cells have been thought to branch off from conventional αβ T cell precursors at the DP stage in the thymus, where the cells in the DP thymocyte pool expressing a rearranged invariant Vα14-Jα18 TCR are positively selected by CD1d^+^ thymocytes [6]–[10]. However, currently available data do not rule out the possibility that NKT cells are derived from a precursor population distinct from that of conventional αβ T cells. Findings in support of independent origins for conventional T and NKT cells include the detection of invariant *Vα14-Jα18* transcripts before thymus formation in fetal (RAG-1-KO×WT)F1 mice, in which the possibility of contamination with maternal NKT cells in the samples tested is formally excluded [11], and the cell surface expression by NKT precursor cells and NKT cells of the granulocyte-macrophage colony stimulating factor (GM-CSF) receptor [12], [13], which is a unique marker for myeloid but not lymphoid lineage cells. Considering their unique characteristics compared with conventional T cells, it seemed possible to us that NKT cell precursors might differ from those of conventional T cells and exist at earlier stages than the DP stage previously defined by fate-mapping studies [8]. To test this possibility, we focused on cells with NKT cell potential at early stages of development, prior to the DP stage in the thymus.

In the present study, we demonstrate that NKT cell precursors are present in DN4 stage thymocytes and have the potential to give rise to mature NKT cells in both *in vivo* and *in vitro* experimental settings. Our findings provide new insights into the early development of NKT cells before expression of the invariant Vα14-Jα18 antigen receptor.

## Results and Discussion

### Invariant *Vα14-Jα18* transcripts are present in the thymic DN4 fraction

**Figure 1:**
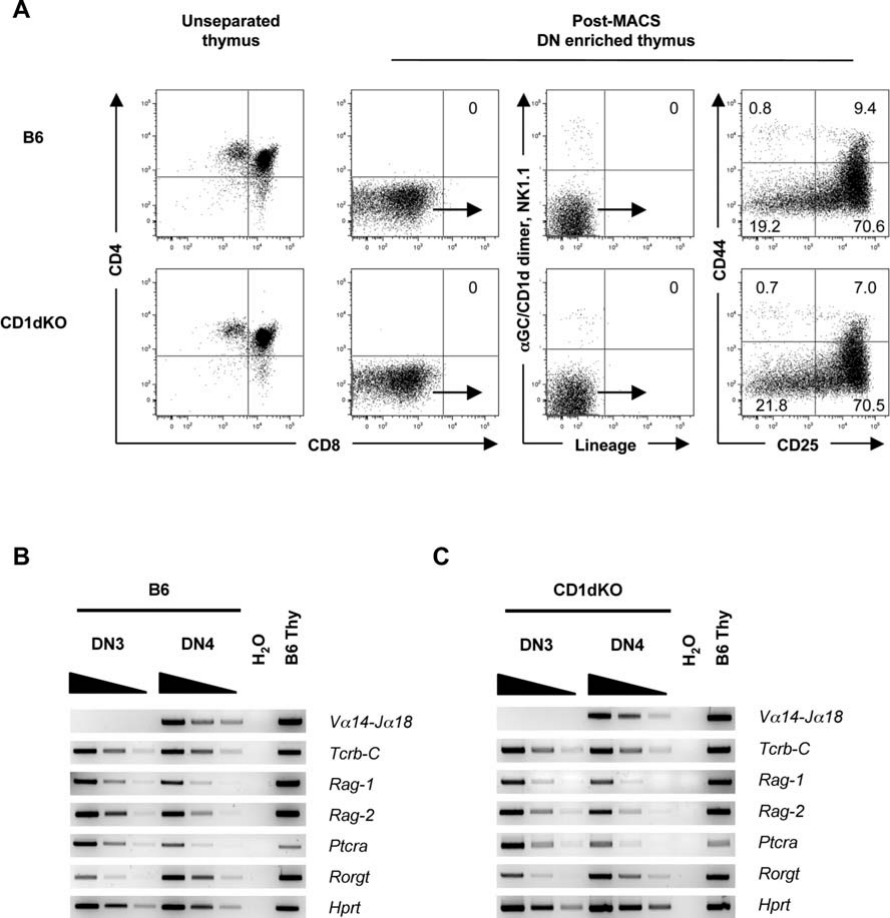
Isolation of the DN4 thymocytes and gene expression analysis. (A) FACS profiles of CD25, CD44 staining of DN thymocytes from B6 and CD1dKO mice. The numbers in FACS plots represent the percentage of cells in the indicated quadrants. DN thymocytes from B6 and CD1dKO mice were enriched by MACS depletion of DP, SP and mature Lin^+^ cell types. αGC/CD1d dimer was used to exclude NKT cells from the DN thymocytes and an extensive lineage mAb ‘cocktail’ (anti-B220, CD3ε, CD11b, CD11c, CD19, Gr-1, TER-119, TCRβ, TCRγδ) was used to remove mature cell types. Dot plots were obtained on a 6-color FACS Aria and are representative of more than six independent experiments for each group of mice. (B and C) Gene expression profiles of the DN3 and DN4 thymic fractions. All mRNA samples of B6 and CD1dKO mice were normalized to the *Hprt* expression level and analyzed by RT-PCR. Horizontal wedges indicate three-fold serial dilutions of cDNA samples. Total thymocytes from B6 mice were used as positive controls. Data are representative RT-PCR results of four independent experiments for each group of mice.

To investigate whether the DN population in the thymus contains cells with NKT cell potential, we first purified Lin^−^ DN3 and DN4 thymocyte fractions devoid of Lin marker positive mature cells, including NKT cells, by a three-step cell purification method. In the first step, the DN fraction was enriched by depletion of DP and SP thymocytes by magnetic-activated cell sorting (MACS) using microbeads coated with anti-CD4 and anti-CD8 monoclonal antibodies (mAbs). Then the DN enriched fraction was stained with a biotin-labeled Lin mAb ‘cocktail’ (anti-B220, CD3ε, CD11b, CD11c, CD19, Gr-1, TER-119, TCRβ, TCRγδ) and subsequently reacted with streptavidin microbeads to deplete Lin^+^ cells using MACS columns. The resulting Lin^−^ cells in the eluate fraction were sorted by staining with fluorescent-conjugated streptavidin and also with directly labeled mAbs (anti-CD4, CD8α, TCRβ, TCRγδ, NK1.1) together with α-galactosylceramide-loaded CD1d (αGC/CD1d) dimer which specifically detects NKT cells [14] to remove any unstained NKT cells and Lin^+^ cells left in the samples. This rigorous cell preparation method and staining protocol was used throughout this study unless otherwise indicated. The Lin^−^ αGC/CD1d dimer^−^ DN fraction was further analyzed by the CD25, CD44 bivariate staining pattern, which defines the well-characterized thymocyte differentiation pathway. As shown in Figure 1A (upper panel), the DN fraction of B6 mice contain neither DP or SP thymocytes, nor αGC/CD1d dimer^+^ NKT cells or Lin^+^ cells. When highly purified DN3 and DN4 fractions from C57BL/6 (B6) mice were analyzed by RT-PCR, the bands corresponding to NKT cell-specific *Vα14-Jα18* sequences were detected in the DN4 but not DN3 fraction (Figure 1B).

Because a fraction of NKT cells in the thymus is DN and CD44^−^, it is still possible that the DN fraction isolated by this method may contain mature or immature NKT cells. To exclude this possibility, we isolated the DN4 fraction from the thymus of CD1d knock-out (CD1dKO) mice, in which NKT cells do not exist because of the lack of expression of CD1d as a selection molecule [15]–[17]. The fluorescence-activated cell sorter (FACS) profiles of the CD1dKO DN fractions were essentially identical to those obtained from B6 mice (Figure 1A, lower panel). Notably, results from the RT-PCR analysis of the CD1dKO DN fractions were identical to those from B6 mice, where the DN4 but not DN3 fraction in both mice expressed NKT cell-specific *Vα14-Jα18* transcripts (Figure 1C). The identities of the *Vα14-Jα18* transcripts detected in the DN4 fractions of B6 and CD1dKO mice were confirmed by direct sequencing of the PCR products (data not shown).

In addition to their expression of invariant *Vα14-Jα18* transcripts, the DN4 fraction of B6 and CD1dKO mice contained TCRβ chain transcripts (Figures 1B and 1C), suggesting that both invariant *Vα14-Jα18* and TCRβ transcripts are already present in the DN4 stage before the cells reach the DP stage. It is important to note that our present findings do not necessarily contradict the previous report, demonstrating that the invariant *Vα14-Jα18* transcripts are detectable at the DP stage [7].

Regarding the possibility of early invariant *Vα14-Jα18* gene rearrangement in the DN population, it has recently been shown that low level of TCRα chain gene rearrangement in the late DN stage, which was previously considered to represent a transgenic artifact, occurs even under physiological conditions [18]. The present findings support the possibility that the DN4 fraction in the thymus contains precursors for the NKT cell lineage.

### Gene expression profile and surface phenotype of the DN4 thymic fraction

To further characterize the DN4 population in the thymus, we used RT-PCR to investigate the expression of genes relevant to the NKT cell development. As shown in Figures 1B and 1C, both B6 and CD1dKO DN4 cells, as well as DN3 cells used as positive controls, expressed transcripts encoding RAG-1/RAG-2, which are required for the rearrangement of TCR gene segments. They also expressed transcripts for pTα (*Ptcra*), which is required for the NKT cell development in a cell autonomous manner [19]. The thymus-specific isoform of retinoic acid receptor-related orphan receptor γ (RORγt) appears to be required for the rearrangement of the *Vα14-Jα18* gene segments as it was shown that RORγt-deficient mice lack *Vα14-Jα18* transcripts and consequently are deficient in NKT cells [8], [10]. As shown in Figures 1B and 1C, RORγt transcripts were clearly detected in the DN4 fraction, and to a lesser extent in the DN3 fraction in both B6 and CD1dKO mice.

Considering that DP thymocytes exclusively express RORγt and using the RORγt-Cre fate-mapping method, it has been concluded that all NKT cells and αβ T cells are derived from DP thymocyte precursors [8]. Our data (Figures 1B and 1C) and those of others [20], [21] clearly demonstrate that RORγt expression begins at the DN3 stage, presumably as a result of pre-TCR signaling. Moreover, the recent report that not all αβ T cells develop through the DP stage [18] supports the possibility that some of NKT cells are not derived from DP precursors.

Next, we analyzed the surface phenotype of the DN4 thymic fraction from both B6 and CD1dKO mice. In contrast to αGC/CD1d dimer^+^ NKT cells in the thymus, which are CD24^low^ CD62L^−^ CD69^+^ and CD122^+^, both B6 and CD1dKO αGC/CD1d dimer^−^ DN4 populations were uniformly CD24^high^, CD62L^+^, CD69^−^, and CD122^−^ (Figure S1). Thus, the DN4 population is different from the αGC/CD1d dimer^+^ NKT cells in terms of cell surface phenotype.

It is well established that the earliest detectable NKT cell precursors just after positive-selection with CD1d in the thymus, which are αGC/CD1d tetramer^+^, have NK1.1^−^, CD4^+^, CD8^+/−^, CD24^+^, CD44^low^ and CD69^+^ surface phenotype [22]. Given our findings that the DN4 fraction lacked expression of the cell surface invariant Vα14 receptor (Figure 1A) and CD69 (Figure S1), but possessed the NKT cell-specific *Vα14-Jα18* transcripts in CD1dKO mice (Figure 1C), we considered that the DN4 population contains the *bona fide* precursors to NKT cells prior to positive-selection with CD1d in the thymus.

### *In vitro* generation of NKT cells from the DN4 fraction

To determine whether the DN4 fraction, which expresses *Vα14-Jα18* transcripts but no cell surface TCR, has NKT cell precursor potential, we co-cultured the DN4 population with OP9 stromal cells engineered to express Notch ligand delta-like 1 (OP9/Dll-1) that support conventional T cell differentiation *in vitro* [23]. Although the developmental requirements of NKT cells might differ from those of conventional T cells, the OP9/Dll-1 stromal monolayer culture system was chosen because of its ability to promote the survival of immature T cells [24].

**Figure 2:**
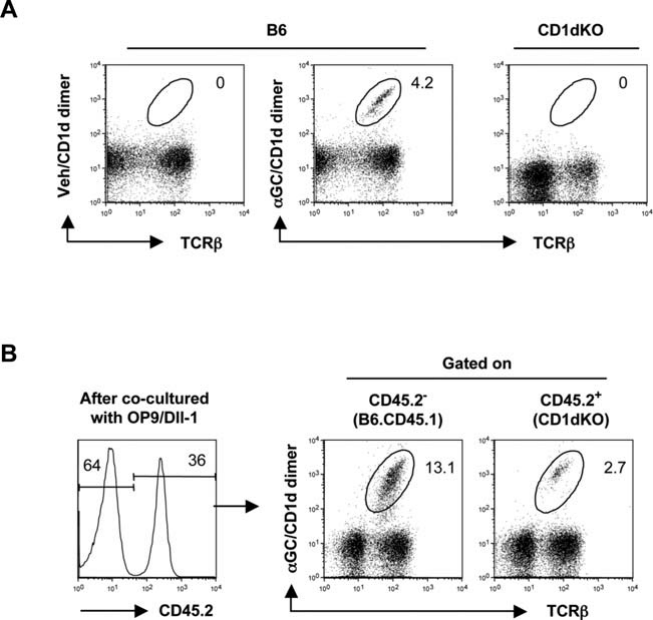
*In vitro* generation of αGC/CD1d dimer^+^ NKT cells from the DN4 thymic population. (A) *In vitro* generation of αGC/CD1d dimer^+^ NKT cells. Thymic DN4 fractions from B6 or CD1dKO mice were co-cultured with OP9/Dll-1 for 8 to 10 days as described in Materials and Methods. The numbers are the percentages of αGC/CD1d dimer^+^ NKT cells within the PI^−^ CD45^+^ viable lymphocyte gate. Representative data from three independent experiments for each group of mice are shown. (B) Mixed co-culture of CD1dKO DN4 with WT DN4 fractions. Thymic DN4 fractions from B6.CD45.1 and CD1dKO (CD45.2^+^) mice were mixed at a ratio of 7∶3, and cultured for 8 days on OP9/Dll-1. Values indicate the percentage of αGC/CD1d dimer^+^ NKT cells within CD45.2^+^ or CD45.2^−^ gated viable lymphocytes. APC-conjugated αGC/CD1d dimer and anti-APC microbeads were used to enrich NKT cells by MACS. Representative data from three independent experiments are shown.

Highly purified (≥99%) DN4 cells from either B6 or CD1dKO mice (Figures 1A and S2) were co-cultured with OP9/Dll-1 in the presence of IL-2, IL-7, and IL-15, as these cytokines are known to have an important role in NKT cell homeostasis [25]. After 8 to 10 days of culture, the DN4 cells from B6 mice, but not those from CD1dKO mice, successfully generated αGC/CD1d dimer^+^ NKT cells (Figure 2A).

To formally exclude the possibility of contamination of our culture system with mature NKT cells and to confirm the presence of NKT cell precursors in the DN4 fraction of CD1dKO mice, we mixed the DN4 fraction from B6.CD45.1 congenic mice with the DN4 fraction from CD1dKO mice at 7∶3 ratio, and then co-cultured with OP9/Dll-1. The CD1dKO DN4 cells, identified as CD45.2^+^, were able to generate αGC/CD1d dimer^+^ NKT cells in the presence of WT CD1d^+^/CD45.1^+^ thymocytes in the 8 day-culture (Figure 2B). These results, together with the data shown in Figure 1C, clearly demonstrated the presence of NKT precursors in the DN4 population. In addition, the results shown in Figure 2B confirmed the requirement for CD1d-expressing thymocytes for the development of NKT cells, in agreement with previous reports [9].

### Phenotype and cytokine secretion profile of NKT cells generated *in vitro* from DN4 thymic fraction

**Figure 3:**
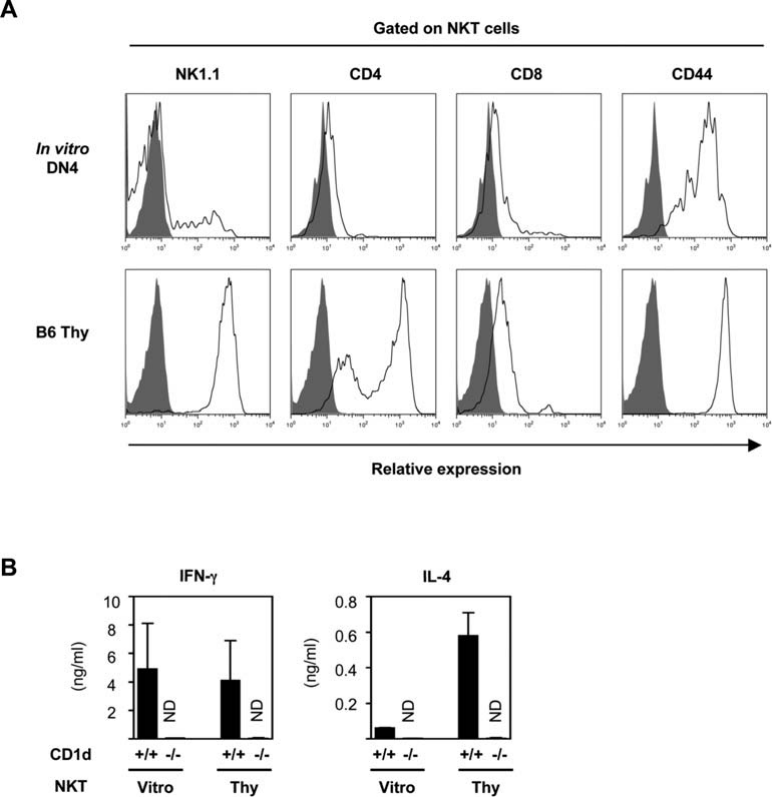
Cell surface phenotype and cytokine production of *in vitro* generated NKT cells. (A) FACS profiles of *in vitro* generated NKT cells. The expression of NK1.1, CD4, CD8 and CD44 antigens by NKT cells generated *in vitro* from B6 DN4 thymocytes on day 10 of culture are compared to those of *ex vivo* B6 thymic NKT cells. Viable αGC/CD1d dimer^+^ NKT cells were gated for the analysis. Representative data from three independent experiments are shown. (B) Cytokine secretion profiles of *in vitro* generated NKT cells. αGC/CD1d dimer^+^ NKT cells generated *in vitro* from B6 DN4 thymocytes cultured on OP9/Dll-1 were FACS sorted on day 10 of culture, as shown in Figure 2A, and co-cultured with αGalCer-pulsed GM-DCs from either B6 or CD1dKO mice. APC-conjugated αGC/CD1d dimer and anti-APC microbeads were used to enrich NKT cells by MACS. The supernatants were collected after 48 hours of culture and were assayed for IL-4 and IFN-γ levels using the CBA method. Freshly sorted B6 thymic NKT cells served as positive controls. Values are expressed as mean±SD from three independent experiments. ND, not detected.

FACS analysis shown in Figure 3A revealed that the most αGC/CD1d dimer^+^ NKT cells generated *in vitro* from the B6 DN4 population were of the NK1.1^−^, DN, and CD44^+^ phenotype, while *ex vivo* B6 thymic NKT cells were of the NK1.1^+^, CD4^+^ or DN, and CD44^+^ phenotype. A standard cytokine secretion assay using α-galactosylceramide (αGalCer)-pulsed GM-CSF-induced bone marrow-derived dendritic cells (GM-DC) demonstrated that the *in vitro* derived NKT cells secreted both IFN-γ and IL-4 in a CD1d-dependent manner, which is the hallmark of NKT cells (Figure 3B). Of note, the cytokine secretion profiles of the *in vitro* generated NKT cells were more of Th1-type, with very little IL-4, compared with their *ex vivo* counterparts (Figure 3B). These differences between *in vitro* generated NKT cells and *ex vivo* thymic NKT cells may be due to the limits of the OP9/Dll-1 culture system, which has been reported to support the preferential development of IFN-γ producing CD8 T cells [23], and may also indicate a requirement for the intact thymic microenvironment for the full differentiation of NKT cells [26]. It was also reported that human DN NKT cells show Th1-type biased cytokine response upon stimulation [27]. Collectively, the data shown in Figures 3A and 3B suggest that the *in vitro* generated NKT cells resemble those found *in vivo*.

### *In vivo* generation of NKT cells from DN4 thymocytes

To further substantiate and confirm our findings in Figure 2B, showing that CD1dKO DN4 population contains cells with NKT cell potential, highly purified DN4 cells from CD1dKO (CD45.2^+^) mice were injected intrathymically into B6.CD45.1-congenic recipients.

**Figure 4:**
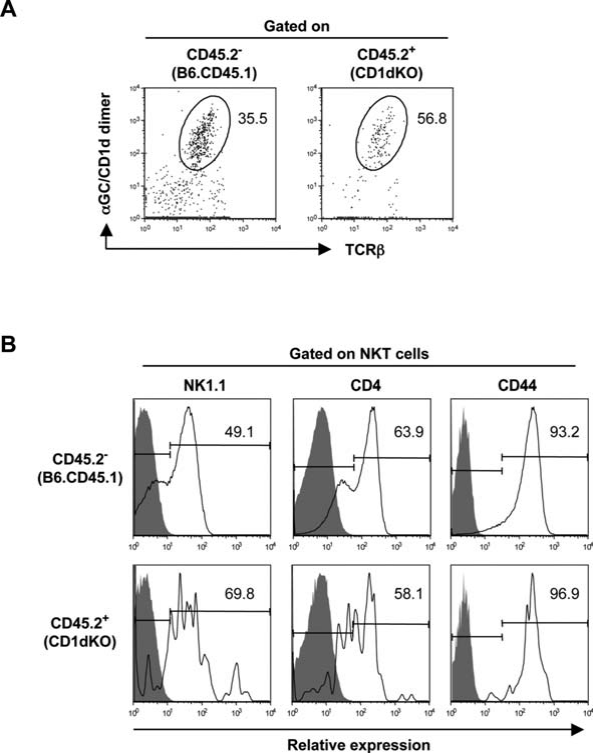
Generation of αGC/CD1d dimer^+^ NKT cells *in vivo* by intrathymic injection of CD1dKO DN4 thymocytes. (A) Detection of αGC/CD1d dimer^+^ NKT cells in the thymus. FACS sorted DN4 thymocytes (6×10^5^) from CD1dKO mice were injected intrathymically into B6.CD45.1 congenic mice, and recipient mice were analyzed 3 weeks later by FACS. Numbers on FACS plots indicate the percentage of cells in the indicated gates. Anti-CD45.2 staining was used to distinguish between cells of donor and recipient origin. APC-conjugated αGC/CD1d dimer and anti-APC microbeads were used to enrich NKT cells by MACS. (B) FACS profiles of αGC/CD1d dimer^+^ NKT cells generated by intrathymic injection of CD1dKO DN4 thymocytes. Histogram plots show the expression of NK1.1, CD4 and CD44 by *in vivo* generated NKT cells from CD45.2^+^ CD1dKO DN4 thymocytes compared with resident NKT cells of CD45.2^−^ B6.CD45.1 host mice. Staining controls are shown in gray. The data is representative of two independent experiments.

As demonstrated in Figure 4A, transfer of CD45.2^+^ CD1dKO-derived DN4 donor cells successfully generated αGC/CD1d dimer^+^ NKT cells in the WT CD45.1 recipient thymus. The surface phenotype of these *in vivo* generated αGC/CD1d dimer^+^ NKT cells from CD1dKO donors resembled that of resident NKT cells in the WT recipients, where the majority of NKT cells were NK1.1^+^ and CD44^+^ (Figure 4B). These results demonstrated that the DN4 thymocytes from CD1dKO mice contained precursors of NKT cells, which is consistent with the presence of the invariant *Vα14-Jα18* transcripts in this population (Figure 1C), and the potential of these cells to give rise to NKT cells when cultured *in vitro* (Figure 2B).

Taken together, we have identified cells in the thymic DN4 population with NKT cell potential in both *in vitro* and *in vivo* experimental settings. Studies to elucidate the differentiation pathway(s) of NKT cells should provide further insights into NKT cell biology and provide clues to solve the puzzles concerning developmental requirements of this unique cell lineage.

## Materials and Methods

### Mice

B6 mice were purchased from CLEA Japan, CD1dKO mice with B6 genetic background were from L. Van Kaer (Vanderbilt University, Nashville, TN) and B6.CD45.1 mice were from The Jackson Laboratory. Mice were maintained under specific pathogen-free conditions in the animal facility of the RIKEN Research Center for Allergy and Immunology and were used at 5–8 weeks of age unless otherwise indicated. All experiments were in accordance with protocols approved by the RIKEN Animal Care and Use Committee.

### Flow cytometry and cell sorting

mAbs specific for B220 (RA3-6B2), CD3ε (145-2C11), CD4 (RM4-5) (GK1.5), CD8α (53-6.7), CD11b (M1/70), CD11c (HL3) CD19 (1D3), CD24 (M1/69), CD25 (PC61), CD44 (IM7), CD45 (30-F11), CD45.2 (104), CD62L (MEL-14), CD69 (H1.2F3), CD122 (TM-b1), Gr-1 (RB6-8C5), NK1.1 (PK136), TCRβ (H57-597), TCRγδ (GL3), TER-119 and isotype controls were purchased from BD Biosciences or eBioscience, and used as FITC, PE, PerCP-Cy5.5, PE-Cy7, APC, APC-Cy7, or Pacific Blue conjugates. αGalCer- or vehicle-loaded soluble dimeric mouse CD1d:Ig fusion protein (BD Biosciences) was used together with APC-anti-mouse IgG_1_ (X56; BD Biosciences) mAb to stain NKT cells. Cell preparation and staining were performed as described [28]. Propidium iodide (PI) (Sigma-Aldrich) was added to the cell suspension immediately before analysis to gate out dead cells where indicated. Flow cytometry was done on a FACS Calibur or FACS Aria (BD Biosciences). Data were analyzed with FlowJo (Tree Star).

DN thymocytes were enriched from total thymocytes by depletion of CD4^+^, CD8^+^ thymocytes using anti-mouse CD4 and CD8 microbeads and MACS LS columns according to the manufacturer's protocol (Miltenyi Biotec). APC-conjugated αGC/CD1d dimer and anti-APC microbeads were used to enrich NKT cells by MACS where indicated. Cells were sorted using FACS Aria. Sorted cells were generally ≧99% pure as determined by post-sort analysis using FACS.

### OP9/Dll-1 co-cultures

OP9/Dll-1 cells were generated by H. Kawamoto and were maintained as described [23]. Sorted DN4 thymocytes (6×10^5^) were plated onto a 10 cm tissue culture plate (BD Falcon) containing a monolayer of OP9/Dll-1 cells seeded at 3.5×10^5^ cells/dish on the previous day. Co-cultures were done in the presence of 5 ng/ml of each IL-2, IL-7 (R&D Systems) and IL-15 (Peprotech). Cells were transferred onto a fresh monolayer on day 5 of culture and analyzed at the indicated time points.

### RT-PCR

Total RNA was prepared from sorted thymocyte populations (5×10^5^) using the RNeasy kit (Qiagen) and treated with DNase (Qiagen). cDNA was synthesized with SuperScript III reverse transcriptase, oligo(dT) and RNaseOut followed by treatment with RNase H (Invitrogen). The list of primer pairs used in RT-PCR is shown in Table S1. The numbers of PCR cycles were as follows: 26 for *Ptcra*; 28 for *Tcrb-C*; 29 for *Hprt* and *Rag-1*; 30 for *Rag-2*; 32 for *Rorgt*; 39 for *Vα14-Jα18*. The amplified products were stained with SYBR Safe DNA gel stain (Molecular Probes) and visualized using a LAS-1000 lumino image analyzer (Fuji Film, Japan). The identity of RT-PCR generated *Vα14-Jα18* bands was confirmed by direct sequencing using an ABI 3100*xl* genetic analyzer.

### Cytokine measurement

GM-DC were prepared from B6 and CD1dKO mice and pulsed overnight with 100 ng/ml of αGalCer. NKT cells (2×10^4^) from either B6 thymus or *in vitro* DN4 culture origin were plated with an equal number of GM-DC in 96-well U-bottom plates (BD Falcon) in a 0.1 ml volume. Culture supernatants were harvested after 48 hours of culture and assayed for IL-4 and IFN-γ levels with cytokine beads array (CBA) Flex Set (BD Bioscience) using a FACS Calibur. Resulting data were analyzed with the FCAP Array software (Soft Flow).

### Intrathymic injection

B6.CD45.1 recipient mice received a slight whole body γ-irradiation (3 Gy) from a cesium source (Gammacell 40). Mice were injected intrathymically with FACS sorted DN4 thymocytes (6×10^5^) in a 10 µl volume using a Hamilton syringe fitted with a 33G needle. Recipient mice were analyzed 3 weeks after injection.

## Supporting Information

Figure S1 Cell surface phenotypes of DN4 thymocytes. Histogram plots show the expression of CD24, CD62L, CD69 and CD122 antigens by DN4 thymocytes gated as shown in Figure 1A from B6 (black) and CD1dKO (blue) mice compared to those of B6 thymic αGC/CD1d dimer^+^ NKT cells (red). 7-color FACS analysis was performed using a FACS Aria. Staining controls are shown in gray. Representative data of three independent experiments are shown.
(1.35 MB TIF)

Figure S2 Post-sort purity of FACS sorted DN4 thymocytes. Post-sort analysis of FACS sorted DN4 thymocytes from B6 and CD1dKO mice. Numbers are the percentage of cells in the indicated quadrants. Representative data from more than six independent experiments are shown.
(0.46 MB TIF)

Table S1 Oligonucleotide sequences for primer pairs used in PCR amplifications.
(0.04 MB DOC)
