# Supplementary figures and images for: Identification of CD4−CD8− Double-Negative Natural Killer T Cell Precursors in the Thymus
Source: PLoS One. 2008 Nov 10;3(11):e3688. doi: 10.1371/journal.pone.0003688 (PMC2577011; doi:10.1371/journal.pone.0003688)

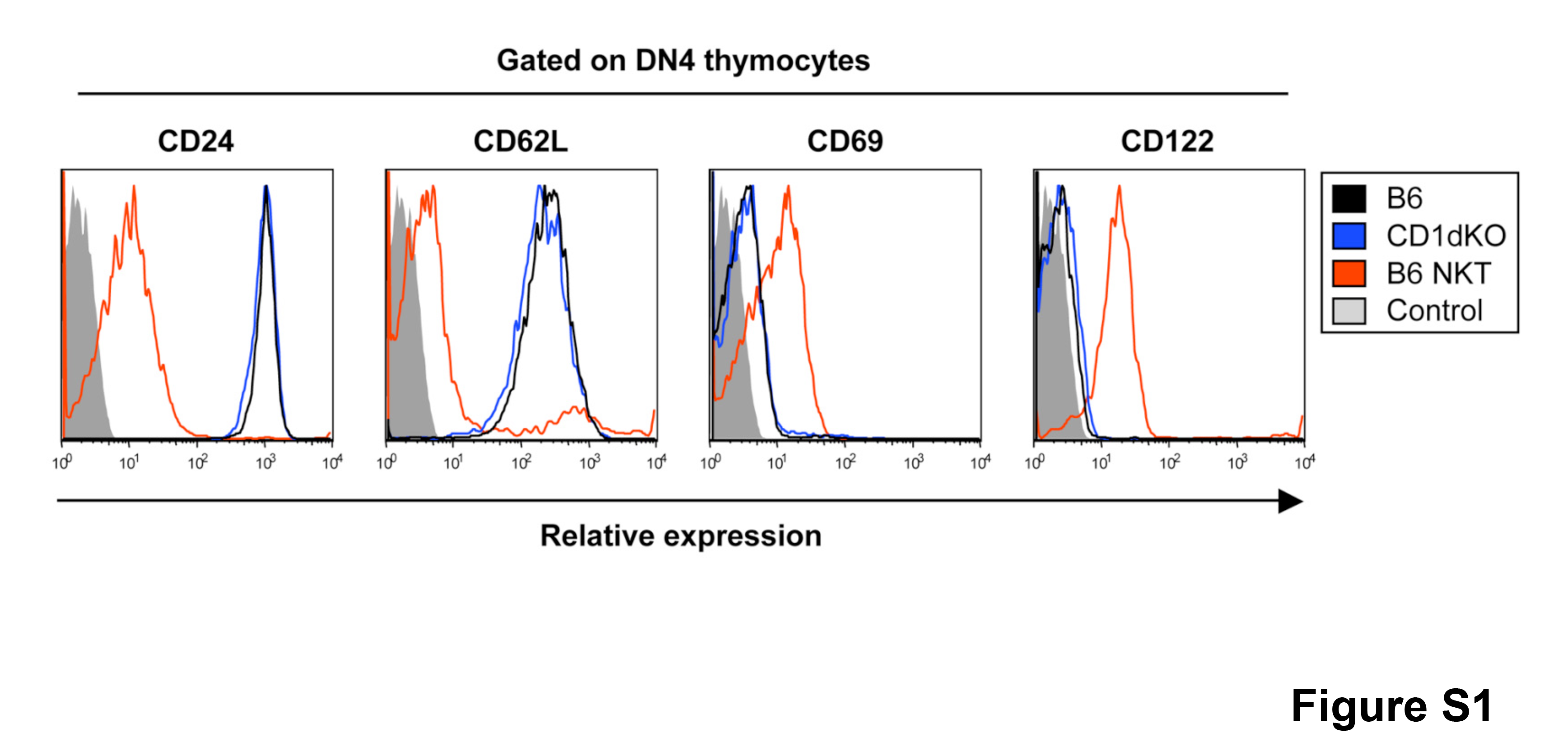

Supplement: Figure S1 — Cell surface phenotypes of DN4 thymocytes. Histogram plots show the expression of CD24, CD62L, CD69 and CD122 antigens by DN4 thymocytes gated as shown in Figure 1A from B6 (black) and CD1dKO (blue) mice compared to those of B6 thymic αGC/CD1d dimer+ NKT cells (red). 7-color FACS analysis was performed using a FACS Aria. Staining controls are shown in gray. Representative data of three independent experiments are shown. (1.35 MB TIF) [file pone.0003688.s001.tif]

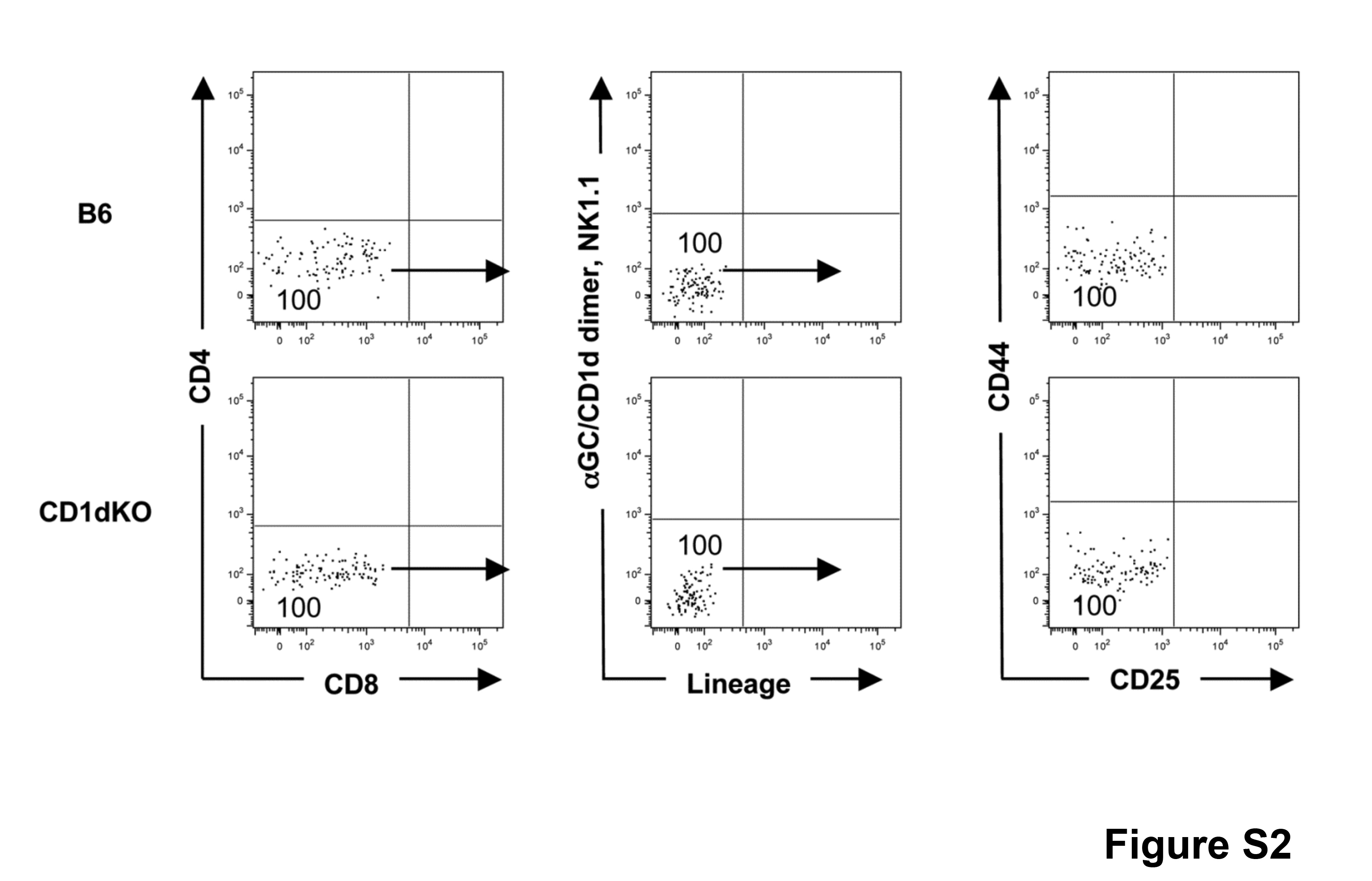

Supplement: Figure S2 — Post-sort purity of FACS sorted DN4 thymocytes. Post-sort analysis of FACS sorted DN4 thymocytes from B6 and CD1dKO mice. Numbers are the percentage of cells in the indicated quadrants. Representative data from more than six independent experiments are shown. (0.46 MB TIF) [file pone.0003688.s002.tif]
