# Supplementary material for: Identification of CD4−CD8− Double-Negative Natural Killer T Cell Precursors in the Thymus
Source: PLoS One. 2008 Nov 10;3(11):e3688. doi: 10.1371/journal.pone.0003688 (PMC2577011; doi:10.1371/journal.pone.0003688)
Supplement: Table S1 — Oligonucleotide sequences for primer pairs used in PCR amplifications. (0.04 MB DOC) [file pone.0003688.s003.doc]

Table S1. Oligonucleotide sequences for primer pairs used in PCR amplifications.

| **Gene** | **Primer sequence** |
| --- | --- |
| Va14 (sense) | 5'-gacccaagtggagcagagtcct-3' |
| Ja18 (antisense) | 5'-cagctccaaaatgcagcctccctaa-3' |
| Ptcra (sense) | 5'-acctccagccaccaccctca-3' |
| Ptcra (antisense) | 5'-tctcaccagcccgacatgcc-3' |
| Rag-1 (sense) | 5'-cccgtgtggcgctcttcatg-3' |
| Rag-1 (antisense) | 5'-gcccaaagggtcccctaagg-3' |
| Rag-2 (sense) | 5'-gtctgtaaccggctactggataacatg-3' |
| Rag-2 (antisense) | 5'-cctgagtctgaggggcttttgc-3' |
| Hprt (sense) | 5'-ctgtgtgctcaaggggggct-3' |
| Hprt (antisense) | 5'-ggactcctcgtatttgcagattcaacttg-3' |
| Tcrb-C (sense) | 5'-caggcctacaaggagagcaa-3' |
| Tcrb-C (antisense) | 5'-catcagcactaggccactga-3' |
| Rorgt (sense) | 5'-ttttgaggaaaccaggcatc-3' |
| Rorgt (antisense) | 5'-ttggcaaactccaccacata-3' |
